# Effect of Intratympanic Dexamethasone on Controlling Tinnitus and Hearing loss in Meniere’s Disease

**Published:** 2014-07

**Authors:** Faramarz Memari, Fatemeh Hassannia

**Affiliations:** 1 *Department of Otorhinolaryngology, Head and Neck Surgery. Iran University of Medical Sciences. Hazrate Rasul Medical Center, Tehran, Iran. *

**Keywords:** Intratympanic dexamethasone, Tinnitus, Fluctuating hearing loss, Meniere’s disease.

## Abstract

**Introduction:**

We investigated the effect of intratympanic dexamethasone on control of tinnitus and hearing loss in patients with Meniere’s disease.

**Materials and Methods:**

100 consecutive patients with a diagnosis of Menier's disease according to the 1995 criteria of The American Academy of Otolaryngology – Head and Neck Surgery (AAO–HNS) who remained symptomatic despite medical therapy were assigned to receive intratympanic dexamethasone. The results were assessed with respect to changes in hearing symptoms and tinnitus.

**Results:**

Hearing improvement and improvement in SDS was observed in 52% and 35% of patients, respectively. Tinnitus score was improved in 57% of patients. There was no relationship between age, sex, duration of disease, unilaterality of disease, or response to therapy.

**Conclusion:**

Intratympanic dexamethasone may be effective in the symptomatic control of hearing loss and tinnitus in Meniere’s disease.

## Introduction

A clinical diagnosis of Menier's disease, as defined by the 1995 American Academy of Otolaryngology – Head and Neck Surgery (AAO–HNS), requires the presence of recurrent, spontaneous episodic vertigo, hearing loss, aural fullness, and tinnitus.

Several studies have demonstrated circulating antibodies and immune complexes in patients with hydrops. Therefore, the routine use of steroids for the treatment of Meniere’s disease has been advocated. Indeed, reduction in immune reactivity with the use of steroids is a part of the treatment protocol for Menier's disease (1). Although the steroids most commonly used for hydrops are glucocorticoids, these agents do have some inherent mineralocorticoid effect that may also play a role in the treatment effects seen with systemic steroid use in these patients (2). 

Intratympanic (IT) steroid injection is used to introduce steroids through the tympanic membrane, resulting in reduced systemic toxicity and a higher perilymph steroid level (3). The purpose of this study was to evaluate the effect of intratympanic steroid injection in the symptomatic control of hearing loss and tinnitus in patients with Meniere’s disease.

## Materials and Methods

This prospective study included 100 consecutive patients with hearing loss and/or tinnitus due to Menier's disease (AAO–HNS criteria) referring to the ear, nose, and throat (ENT) clinic at Hazrate Rasul Medical Center. Patients with persistent hearing loss and/or tinnitus despite maximum medical therapy (including dietary modification, diuretics, and vasodilators) for a minimum of 1 month were included. 

Approval of the Ethics Committee of the Department and Research Center of Otolaryngology and Head and Neck surgery was obtained. All patients gave their informed consent prior to their inclusion in the study. 

Patients with tinnitus were scored using the tinnitus handicap inventory (THI; Newman et al. 1996) before and after treatment. 

For all 100 patients, standard treatment was a slow intratympanic injection of 0.4–0.5 ml dexamethasone (4mg/ml). The procedure was performed in a semi-supine position under a microscope. 

Using a 27-gauge spinal needle and 2-ml syringe, a puncture was made at the anteroinferior portion of the tympanic membrane. The patient was instructed to avoid swallowing with his/her head tilted 45º to the healthy side for 30 minutes. IT steroid injections were administered weekly for 3 consecutive weeks. PTA, SDS, and tinnitus score was obtained immediately prior to the first injection and was repeated 3 weeks after the final injection.

Hearing improvement was defined as an improvement in the PTA of ≥10 db (average of three frequencies at 500, 1,000, and 2,000 Hz) Statistical analysis was performed using the paired t test. 

## Results

The average age of the patients was 41 years, and the male to female ratio was 1.4:1.37. Sixty-two patients had bilateral involvement and seven had diabetes mellitus. Descriptive data of the patients are presented in (Table 1).

The initial PTA level was >20 db in 84 patients. PTA hearing improvement of approximately ≥10 db over three consecutive frequencies was noted in 44 (52%) of 84 patients. Seven (8%) patients showed improvements >20 db in PTA (Table 2).

Seventy-three of 100 patients had abnormal SDS, with improvement in SDS observed in 26 (35%) of these cases. Eighty-seven of 100 patients complained of tinnitus, while 49 (57%) of these patients showed improvement in their tinnitus score. Tinnitus score was not worsened in any patient. 

**Table 1:** Descriptive data

	**N**	**Minimum**	**Maximum**	**Mean**
Age	100	21	66.00	41.5000
PTA^1^	100	.00	90.00	44.4100
SDS 1	100	60.00	100.00	77.8100
Tinnitus 1	100	.00	88.00	40.5500
PTA 2	100	.00	90.00	37.0300
SDS 2	100	60.00	100.00	89.4600
Tinnitus 2	100	00	88.00	31.3000

1 = Pre injection;

2 = Post injection

**Table 2:** Hearing improvement

**No gain**	**<10 db hearing gain**	**>20 db hearing gain**	**>10 db hearing gain**	**Total**
40(48%)	16(19%)	7(8%)	21(25%)	84

The mean values of PTA, SDS, and tinnitus score before treatment were 44 db, 77% and 40 respectively; changing to 37 db, 89% and 31, respectively following treatment (P<0.05). 

The average age was 40 years in the responsive group and 42 years in the non-responsive group. Male to female ratio in the responsive group and non-responsive groups were 21/18 and 25/17, respectively. 

There was no significant difference in age (P=0.508), sex ratio (P=0.525), time of onset to therapy (P=0.158), initial SDS level (P= 0.615), or unilaterality of disease (P=0.170) between responders and non-responders (Tables 3–5).

**Table 3:** Comparison of the two groups according to PTA improvement

	**Responders**	**Non responders **
Number	44	40
Age	41	43
Sex (M/F)	27/25	25/23
Initial PTA	(SD=20.99)52	(SD=21.10)43

**Table 4:** Comparison of the two groups according to SDS improvement

	**Responders**	**Non-responders**
Number	26	47
Age	38	43
Sex (M/F)	14/12	28/19
Initial SDS	(SD=10.56) 83%	(SD=10.71) 83%

**Table 5:** Comparison of the two groups according to tinnitus score improvement

	**Responders**	**Non responders**
Number	49	38
Age	42	42
Sex (M/F)	26/31	23/20
Initial tinnitus score	(SD=20.86) 52	(SD=21.74) 41

In Table 6 we can also see that most of the non-responders initially had higher PTA levels.

There was no significant difference in response to therapy between diabetic and non-diabetic patients.

In this study the only complication of IT injection was temporary dizziness in some patients, and no patients developed tympanic membrane perforation or otitis media.

**Table 6:** Hearing improvement according to PTA before treatment

	>20 db hearing gain	<20 db hearing gain	No gain
26–40 db (n=28)	1(3.5%)	7 (28%)	20 (78%)
41–55 db (n=35)	1(2.8%)	24 (68%)	10 (28%)
56–70 db (n=13)	3(23%)	6 (46%)	4(30%)
71–90 db (n=8)	2(25%)		6(75%)

## Discussion

Immunologic injury is implicated in many inner ear pathologies, and Meniere’s disease may be due in some cases to immune dysfunction. Immunologic or allergic causes of Meniere’s disease were proposed as early as the 1890s. In a recent study conducted by Tomoda et al. (3), 30 patients with classic Meniere’s disease underwent systemic and otologic investigations. Several cases showed hypergammaglobulinemia and antibody elevation to type 2 collagen in the serum and endolymph. Five of 18 patients (28%) were treated with oral prednisolone (60 mg/day). Hughes et al. also reported a good response in 20% of Meniere’s patients treated with oral prednisolone (4).

Shea reported that Meniere’s patients with acute rapidly progressive hearing loss have a marked response to oral dexamethasone (5).

Recently, intratympanic (IT) steroids have been used more widely due to lack of systemic side effects. 

IT steroids may have an anti-inflammatory effect in the labyrinth, as suggested by the beneficial response in inner ear diseases with likely immune causes (6). In addition, recent *in vitro* studies suggest that steroid perfusion of labyrinthine tissues can affect sodium and fluid transport (7). 

Steroids are often used in Meniere’s disease treatment protocols. Itoh and Sakata reported the first IT steroid protocol in 1987, in which four to five weekly injections of 2 mg of dexamethasone were administered to 61 patients with unilateral Meniere’s disease. This protocol resulted in relief of vertigo in 80% of patients and reduction of tinnitus in 74% of patients (8). In an initial study, Shea and Ge used a combination of intratympanic and intravenous corticosteroids to produce a 67.9% improvement in hearing and a 96.4% control of vertigo in 28 patients who had varying severities of Meniere’s disease (1). 

Shea later reported the 2-year results of 48 Meniere’s disease patients who showed a 35% improvement in hearing and a 63% control of vertigo (9).

Silverstein et al. showed no hearing improvement in a prospective double-blind randomized crossover study (10). In a recent presentation, Hamid used 24 mg/ml dexamethasone, with 2-year results showing vertigo control and hearing improvement in 90% of 60 patients with Meniere’s disease, without significant side effects (11).

In 2003, Hillman Todd et al. evaluated the effect of IT dexamethasone in 50 patients with Menier's criteria. Hearing improved acutely in 40% of patients, deteriorated in 4%, and did not change in 56%. The average decrease in threshold was 14.2 db (2).

In 2004, Selivanova et al. showed that IT combined dexamethasone/hayaluronic acid application provides a reliable therapeutic option for improvement of hearing in Meniere’s patients who have failed intravenous steroid treatment (12).

Variation in reported studies in terms of treatment schedules, medications, method of administration, and dosing frequencies have made it difficult to assess the efficacy of IT steroids in the treatment of Meniere’s disease. In addition, analysis of any therapy in Meniere’s disease is associated with several obstacles: the etiology of the syndrome is unknown; there is no cure; the therapy is empirical; there is no clear endpoint for therapeutic success; and follow up is difficult (13). The natural history of Meniere’s disease, which is a fluctuation in symptoms and overall unpredictable course, makes analysis of treatment outcomes even more complex. 

Despite these mixed preliminary results, use of IT steroid therapy appears to be increasing in clinical practice. This is likely influenced by the convenience and ease of repeating these office-based treatments. Moreover, minimal side effects have been reported in relation to IT steroid therapy (14). 

The only complication of IT injection in our study was temporary dizziness in some patients, while no cases of tympanic membrane perforation or otitis media were observed.

In this study, hearing improvement was noted in 44 (52%) of 84 patients, while seven (8%) patients showed >20 db hearing gain. Based on these observations, it cannot be ruled out that our results were simply a placebo effect or the natural fluctuation of symptoms. On the other hand, the fact that these patients had persistent hearing loss and tinnitus and no fluctuation in their hearing in the month before their IT injection (despite receiving medical therapy) suggests that their improvement could be a response to IT steroids. Further, there was a high initial response as opposed to the longer-term resolution noted in the natural history of the disease, and this would seem to give some credence to the efficacy of the IT therapy. 

Correlations of the various factors to response to treatment were evaluated. There was no significant difference in age, sex, time of onset to treatment, unilaterality or response to treatment. There was a significant correlation between initial PTA and initial tinnitus score and response to treatment. 

In total, 7% of our patients were diabetic. We did not observe any significant differences between diabetic and non-diabetic patient in response to therapy. 

The lack of a control group is one limitation of this study. However, a placebo-controlled study would not be ethically acceptable, although use of an active control group would be possible.

## Conclusion

Intratympanic dexamethasone injection could be a simple and effective treatment for the symptomatic control of hearing loss and tinnitus in patients with Menier's disease. However, further double-blind, controlled studies with larger numbers of patients are needed to confirm these results.

## References

[1] Shea JJ Jr, Ge X (1996). Dexamethasone perfusion of the labyrinth plus intravenous dexamethasone for Meniere’s disease. Otolaryngol Clin North Am.

[2] Hillman Todd M, Arriaga Moises A, Chen Douglas A (2003). Intratympanic steroids: do they acutely improve hearing in cases of cochlear hydrops?. Laryngoscope.

[3] Tomoda K, Suzuka Y, Iwai H, YamashitaT, Kumazawa T (1993). Menier’ disease and autoimmunity: clinical study and survey. Acta otolaryngol suppl.

[4] Houghes GB, Kinner SE, Barna BP, Calabrese LH (1984). Practical versus theoretical management of autoimmune inner ear disease. Laryngoscope.

[5] Shea JJ (1983). Autoimmune sensorineural hearing loss as an aggravating factor in Meniere’s disease. Adv Otorhinolaryngol.

[6] ParnesLS, Sun AH, Freeman DJ (1999). Corticosteroid pharmacokinetics in the inner ear fluids: an animal study followed by clinical application. Laryngo- scope.

[7] Pondugula SR, Sanneman JD, Wangemann P, Milhaud PG, Marcus DC (2004). Glucocorticoids stimulate cation absorption by semicircular canal duct epithelium via epithelial sodium channel. American Journal Physiol Renal Physiol.

[8] Itoh A, Sakata E (1991). Trearment of vestibular disorders. Acta Otolaryngo.

[9] Shea JJ Jr (1997). the role of dexamethasone or streptomycin perfusion in the treatment of Meniere’s disease. Otolaryngol Clin North Am.

[10] Silverstein H, Isaacson JE, Olds MJ, Rosenberg S (1998). Dexamethasone inner ear perfusion for the treatment of Meniere’s disease: a prospective, randomized, double-blind, crossover trial. Am J Otol.

[11] Hamid MA (2001). Intratympanic dexamethasone perfusion in Meniere’s disease. spring meeting of the American Neurotology Society.

[12] Selivanova Oksana A, Gouveris H, Victor A, Amedee R, Mann W (2005). Intratympanic dexamethasone and hyaluronic acid in patients with low frequency and Menier's- associated sudden sensorineural hearing loss. Otology and neurotology.

[13] Hamer SG, Driscoll CLW, Facer GW, Beatty C W, McDonald TJ (2001). Long term follow up of ranstympanic gentamicin for Meniere’s syndrome. Otology and Neurology.

[14] Soledad Boleas-Aguirre M, Lin FR, Della Santina C, Minor L, Carey J (2008). Longitudinal results with intratympanic dexamethasone in treatment of Meniere’s disease. Otology and Neurotology.

