# Correction: Effect of Intratympanic Dexamethasone on Controlling Tinnitus and Hearing loss in Meniere’s Disease

**Published:** 2014-10

Faramarz Memari,Fatemeh Hassannia. Effect of Intratympanic Dexamethasone on Controlling Tinnitus and Hearing loss in Meniere’s Disease. Iran J Otorhinolaryngol. 2014;26(76):129–133.

The correct title is provided. On the title and text, Menier’s should be Meniere’s.

